# Gut microbiome and resistome characterization of pigs treated with commonly used post-weaning diarrhea treatments

**DOI:** 10.1186/s42523-024-00307-6

**Published:** 2024-05-03

**Authors:** Judith Guitart-Matas, Maria Ballester, Lorenzo Fraile, Laila Darwich, Noemí Giler-Baquerizo, Joaquim Tarres, Sergio López-Soria, Yuliaxis Ramayo-Caldas, Lourdes Migura-Garcia

**Affiliations:** 1 Joint Research Unit IRTA-UAB in Animal Health, Animal Health Research Centre (CReSA), Autonomous University of Barcelona (UAB), Catalonia, Spain; 2 Institute of Agrifood Research and Technology (IRTA), Animal Health Program (CReSA), WOAH Collaborating Centre for the Research and Control of Emerging and Re-Emerging Swine Diseases in Europe, Autonomous University of Barcelona (UAB), Catalonia, Spain; 3 Animal Breeding and Genetics Program, Institute of Agrifood Research and Technology (IRTA), Catalonia, Spain; 4 School of Agrifood and Forestry Science and Engineering (ETSEA), Department of Animal Production, University of Lleida, Catalonia, Spain; 5 Department of Animal Health and Anatomy, Autonomous University of Barcelona (UAB), Catalonia, Spain

**Keywords:** Microbiome, Metagenomics, Post-weaning, Swine, Resistome, Antimicrobial resistance

## Abstract

**Background:**

The global burden of antimicrobial resistance demands additional measures to ensure the sustainable and conscious use of antimicrobials. For the swine industry, the post-weaning period is critical and for many years, antimicrobials have been the most effective strategy to control and treat post-weaning related infections. Among them, post-weaning diarrhea causes vast economic losses, as it severely compromises piglets’ health and growth performance. In this study, 210 piglets were transferred from a farm with recurrent cases of post-weaning diarrhea to an experimental farm and divided into six different treatment groups to determine the effect of the different treatments on the growth performance and survival, the microbiome, and the resistome in a cross-sectional and longitudinal study. The different treatments included antimicrobials trimethoprim/sulfamethoxazole, colistin, and gentamicin, an oral commercial vaccine, a control with water acidification, and an untreated control. An extra group remained at the farm of origin following the implemented amoxicillin routine treatment. A total of 280 fecal samples from pigs at four different sampling times were selected for metagenomics: before weaning-treatment at the farm of origin, and three days, two weeks, and four weeks post-treatment.

**Results:**

The control group with water acidification showed a reduced death risk in the survival analyses and non-significant differences in average daily weight gain in comparison to the antibiotic-treated groups. However, the growth-promoting effect among antibiotic-treated groups was demonstrated when comparing against the untreated control group at the experimental farm. After four weeks of treatment, diversity indexes revealed significantly decreased diversity for the untreated control and the group that remained at the farm of origin treated with amoxicillin. For this last group, impaired microbial diversity could be related to the continuous amoxicillin treatment carried out at the farm. Analysis of the resistome showed that both gentamicin and amoxicillin treatments significantly contributed to the emergence of resistance, while trimethoprim/sulphonamide and colistin did not, suggesting that different treatments contribute differently to the emergence of resistance.

**Conclusions:**

Overall, this shotgun longitudinal metagenomics analysis demonstrates that non-antibiotic alternatives, such as water acidification, can contribute to reducing the emergence of antimicrobial resistance without compromising pig growth performance and gut microbiome.

**Supplementary Information:**

The online version contains supplementary material available at 10.1186/s42523-024-00307-6.

## Introduction

The emergence of antimicrobial resistance (AMR) is an increasing concern for global health. Statistical models have estimated almost 5 million deaths associated with AMR in 2019, including over 1.2 million deaths attributable to AMR of bacterial origin [1, 2]. The rapid rise in AMR has been correlated with the excessive and indiscriminate use of antimicrobials, not only in humans, animals, and plants, but also in food, feed production, and the environment. In recent last decades, the European Union (EU) has implemented different regulations on veterinary medicine with the aim of reducing the overuse of antimicrobials in livestock and ensuring sustainable and responsible use of antibiotics in animal husbandry. However, as antimicrobials are still required to control infectious diseases, other measures to protect health systems, such as the development of rapid diagnostic tests, the implementation of infection prevention and control measures, or the exploration of other alternatives such probiotics, must be further investigated.

Within the swine production sector, weaning is one of the most critical periods. At this phase of the rearing cycle, a relevant disease for the swine industry worldwide is post-weaning diarrhea (PWD). Generally, in conventional farms, 21-to-28-day-old piglets are separated from their mothers and transferred to a different environment. At this point, maternal antibodies start to decrease, the diet abruptly changes to solid feed, and litters are mixed, occasionally causing fights for their hierarchies with the rest of new pen mates. All these stressors severely affect the intestinal health, growth, and wellbeing of piglets. Additionally, PWD is a multi-factorial disease driven by external and predisposing factors, such as age and weight at weaning, genetic susceptibility, and pre-weaning health [3–5]. PWD is commonly associated with enterotoxigenic *Escherichia coli* (ETEC) proliferation, although other bacteria may follow, such as *Salmonella* spp., *Clostridium perfringens* type A, the parasite *Cryptosporidium*, and enteric viruses, such as rotavirus and coronavirus strains [6, 7]. Consequently, gut microbiota disturbances enable pathogen colonization and trigger pro-inflammatory responses that result in distinct PWD symptomatology, characterized by profuse diarrhea, dehydration, anorexia, slow growth, and ultimately death [8, 9]. Over the years, antimicrobials have been traditionally prescribed to reduce the economic losses caused by PWD during this transition period. Among them, colistin has been broadly used to control PWD for its efficacy and reduced cost [5]. However, considering it is a last resort antibiotic, the emergence of colistin-resistant strains raised a major concern to public health, that led to investigate onto other non-antimicrobial strategies. Some examples are the use of organic acids, prebiotics, probiotics, vaccines, bacteriophages, spray dried plasma, antibodies, and antimicrobial peptides [10–16].

AMR bacteria raise a relevant concern that must be considered when implementing an antibiotic treatment on the farm [17, 18]. Recent published legislation prohibited the routine and preventive usage of antimicrobials in animal husbandry and banned supplementing the feed with high doses of zinc oxide [19]. Despite being effective in reducing PWD and mortality and enhancing growth in pigs, zinc oxide pollutes the soil with heavy metals and favors the emergence of AMR bacteria, such methicillin-resistant *Staphylococcus aureus* [20–22]. Besides, different approaches have been tested to prevent the emergence of PWD, such as the use of breeds with slow growth, higher weaning age of piglets, inclusion of diets that promote gut health, and reduction of stocking density [23–25]. However, when animals present clinical signs of the disease, the treatment with antimicrobials is essential. Generally, antimicrobials are prescribed empirically (i.e., not based on the resistance profile of the target pathogen), but the veterinarian may also prescribe the antibiotic depending on the resistance profile of the *E. coli* involved, starting with antimicrobials categorized as D (prudent use), followed by C (caution) or B (restriction). This practice is becoming more common in Spain to fulfill the European requirements for the prudent use of antimicrobials, as published by Vilaró et al., 2020 [26]. Common antimicrobials treatments include ampicillin, amoxicillin, apramycin, neomycin, tetracyclines, trimethoprim/sulphonamide, spectinomycin, gentamicin, and cephalothin or ceftiofur [27, 28]. However, little is known about the effect of the different antimicrobial families on the abundance and diversity of microbial communities and their antimicrobial resistance profiles in the gut or the long-term effect during the transition period [29–31]. In this study, we analyzed the longitudinal fecal microbiome and the resistome of piglets selected from a farm with recurrent problems of PWD using shotgun metagenomic sequencing. The investigation was conducted in an experimental farm where animals were transferred and divided into different treatment groups. Sampling was performed at pre- and post-weaning stages, corresponding to pre- and post-treatment points. Hence, the main objectives of this study were to assess the effect of different treatments commonly used to treat PWD on the growth performance and survival, and the microbial diversity, composition and resistome. Different treatment groups and sampling timepoints were also compared to perform a cross-sectional and longitudinal study.

## Materials and methods

### Animal and experimental design

**Fig. 1:**
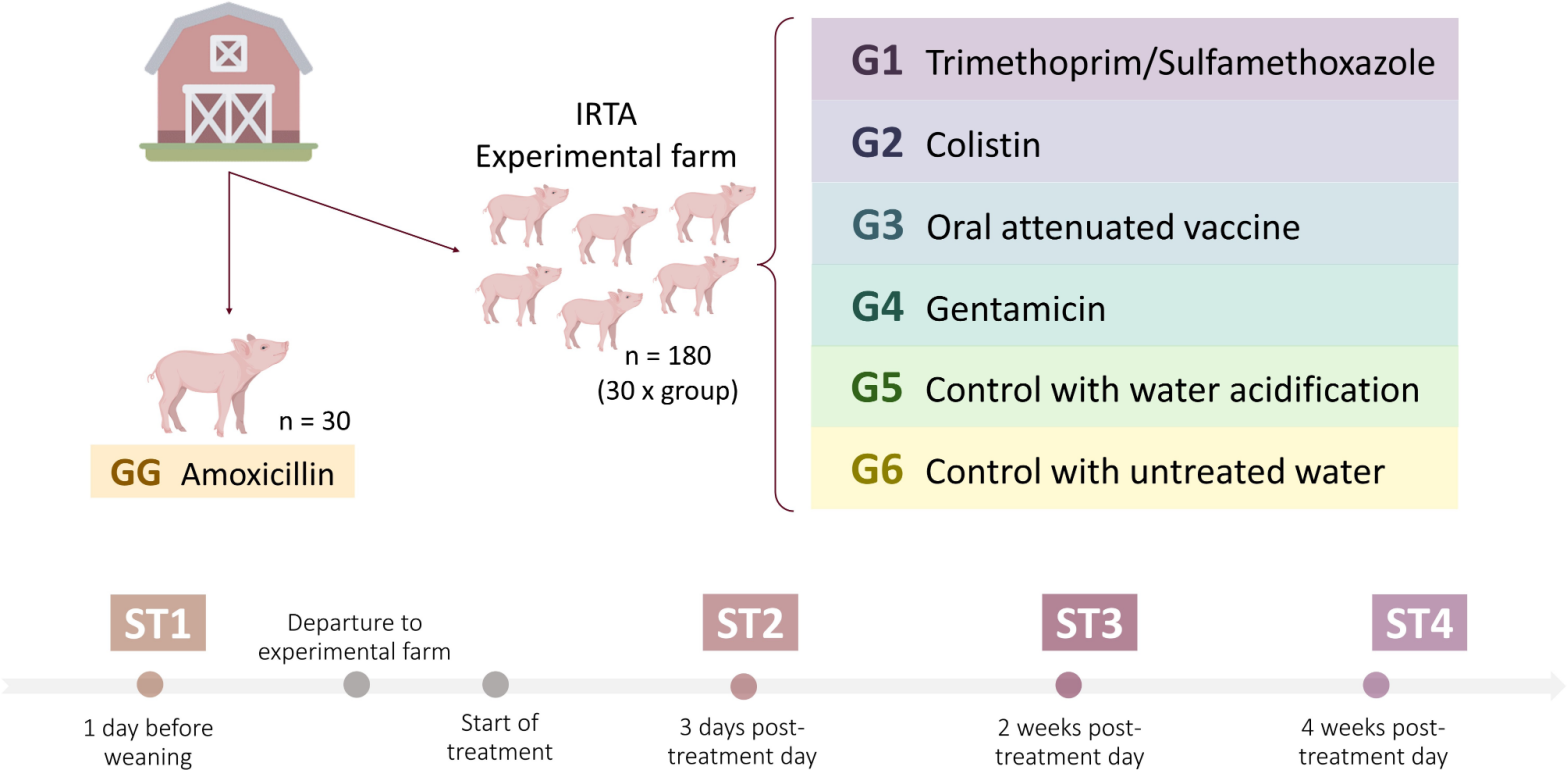
Experimental design including the seven different treatment groups and fecal sampling times

A total of 30 sows without previous records of antimicrobial consumption were randomly selected from a farm with recurrent problems of PWD located in Catalonia (Spain) in October 2020. After farrowing, 7 piglets per sow were ear tagged up to a total of 210 piglets. None of them were treated with antimicrobials during the nursery period. After weaning, 20-day-old piglets were divided into seven treatment groups including one sibling per sow in each of the different treatments. One group remained at the farm of origin (GG), which implemented a routine program of amoxicillin treatment. On this farm, amoxicillin was used prophylactically after weaning due to the high prevalence of *Streptococcus suis* associated diseases (meningitis and arthritis). This treatment was decided by the responsible veterinarian after trying alternative measures such as medium-chain fatty acids in the feed, and other management measures (maintaining litter integrity). The remaining piglets were transferred to an experimental farm from the *Institut de Recerca i Tecnologia Agroalimentàries* (IRTA) and were divided into six different treatment groups: trimethoprim/sulfamethoxazole (G1), colistin (G2), commercial oral *E. coli* vaccine (G3), gentamicin (G4), untreated control with water acidification (G5), and untreated control (G6) (Fig. 1).

Antibiotic treatments were selected based on previous epidemiological data from the farm and after the isolation of *E. coli* from previous clinical cases and the determination of minimal inhibitory concentrations (MICs) against 12 antibiotics as previously published [13]. All antibiotics were applied orally in water for five days when individual animals from different groups showed clinical signs of mild diarrhea. The signs started eleven days after arrival at the experimental farm. Dosages and concentrations were determined by the summary of product characteristics (SPC) of Methoxasol for the trimethoprim/sulfamethoxazole treatment (25 mg/kg/day, Genera Inc.), Apsasol for the colistin treatment (1.5 × 10^5^ international units (IU)/kg/day, Andrés Pintaluba, S.A.), and Gentavet for the gentamicin treatment (2 × 10^3^ IU/kg/day, Fatro S.p.A.). The commercial lyophilizate vaccine (Coliprotec F4/F18, Elanco GmbH) included nonattenuated and nonpathogenic *E. coli* O8:K87 and O141:K94. It was applied orally in a single dose the day of arrival at the experimental farm. For the acidification of the drinking water, phosphoric acid 75% was used (Serbonet Reductor pH, Inserbo, S.L.) from the arrival of the animals at the experimental farm until the end of the study.

The IRTA experimental farm was decontaminated, cleaned, and disinfected before piglets’ arrival. Biosecurity measures were taken during the whole experiment to prevent cross-contamination between groups. The feed provided to the animals at the experimental farm was supplied by the producer and was as in the farm of origin.

Once at the experimental farm, clinical signs were monitored daily, and piglets’ weight was measured at the beginning and at the end of the experiment and compared for significant differences between litters and treatment groups. Sick animals were transferred to the nursery and treated with ceftiofur. Postmortem examination was carried out by an experienced swine veterinarian. From some dead animals, isolation of the pathogen was carried out and antimicrobial susceptibility testing was performed using the microdilution method for minimal inhibitory concentration as described by Vilaró et al., 2022 [32].

Fecal samples were collected from individual piglets on four different occasions: at the farm of origin 1 day before departure to the experimental farm (ST1), 3 days post-treatment (ST2), 2 weeks (ST3) and 4 weeks (ST4) post-treatment. Time-lapse from the last three sampling time points refers to the first treatment day approximately eleven days after arrival at the experimental farm, when clinical signs appeared (Fig. 1).

### Ethical statement

Animals in the experimental farm were exposed to the same conditions as in the conventional farm and were allocated following legislation in animal welfare. Antimicrobial treatments followed the SPC of the products, and no disease was induced. The Ethics Committee for Animal Experimentation (CEEA) guidelines reviewed and authorized the procedures of this study with the ID number CEEA103/2018.

### DNA extraction and whole-metagenomic sequencing

Ten animals per group were selected, excluding those animals transferred to the nursery at some point during the experiment treated with ceftiofur. The DNA from the 280 fecal samples was extracted using the QIAamp® PowerFecal® Pro DNA Kit (Qiagen) according to the manufacturer’s instructions with an elution volume of 50 µl. DNA was quantified using the Qubit dsDNA Broad Range assay kit (Thermo Fisher Scientific). All extracted bacterial DNA was paired end sequenced (2 × 150 bp) for shotgun metagenomics on an Illumina NovaSeq 6000 platform at an 8–10 Gb sequencing depth (Novogene Bioinformatics Technology). For detailed information about animals selected for metagenomic sequencing see Additional File: Table S1.

### Bioinformatic analyses

Metagenomic sequencing reads were filtered with KneadData software to conduct quality control. This software performed a trimming step using Trimmomatic v0.39.2 to remove adapter sequences and reads shorter than 50 bp. It also excluded reads with an average Phred score under 20 in a four-base sliding window [33]. The host-decontamination step was also performed with this software based on Bowtie2 v2.4.4 to separate swine genome sequences (*Susscrofa*11.1 assembly) with the options “--sensitive” and “--dovetail” [34]. FastQC v0.11.9 and MultiQC v1.13 were used to evaluate read quality before and after trimming and decontamination [35, 36]. Kraken2 software was chosen for taxonomic assignation of the remaining reads against the maxikraken2_1903_140GB database at a 0.01 confidence score [37].

### Microbial diversity and resistome characterization

The R microeco package, based on the R6 class system, was used for data processing [38]. Taxonomy reports generated by Kraken2 with a MetaPhlAn (mpa)-style were combined to create abundance and taxonomy tables required for microeco input. Sample metadata were also included in the microtable-class object. The final dataset was trimmed with the “*tidy_dataset*” function to clean empty rows and columns of the dataset. Before the estimation of the alpha diversity indexes, samples were rarefied at a depth of 665,830 reads to correct for the sequencing depth. Beta diversity indexes were also measured with this software. Microbial diversity indexes were plotted with “*ggplot”*, and taxonomy was represented at the phyla and genera levels with the Phinch framework [39].

Characterization of antimicrobial resistance profiles was performed using Resfinder v4.2.5 [40] from quality trimmed reads. This software allowed the identification of acquired antimicrobial resistance genes (ARGs) against the Resfinder database and their quantification by the addition of the “*--out_json*” flag in the Resfinder script. From the output JSON file obtained per sample, sequencing depth of each identified ARG was extracted and normalized by the total number of each set of PE reads per sample, calculated in parts per million (PPM) and summed per antibiotic class for further comparison statistical analyses. For specific formulas performed from Resfinder JSON output to normalize ARG abundances per gene and antibiotic class see Additional File: Tables S2 and S3.

### Statistical analyses

All statistical analyses were performed in R version 4.1.2 [41]. Piglet initial weight was compared between treatment groups to verify that no differences in weight were observed before treatment between groups transferred to the experimental farm. The average daily weight gain (ADWG) measured at the end of the experiment was also compared between treatment groups. After checking for normality of the data using Shapiro-Wilk’s method [42], pairwise comparisons between treatment and control groups were performed, and *P*-values were adjusted using the Benjamini-Hochberg procedure to control the false discovery rate [43]. Proportions of sick and dead animals were also compared between treatment groups at the end of the experiment using a proportional hazards model for survival analyses [44].

Statistical differences in microbial diversity were evaluated using the Shannon index obtained from microeco, which accounts for diversity richness and evenness [45]. To study homogeneity within treatment groups and sampling times, Kruskal-Wallis nonparametric one-way analysis of variance was performed. The nonparametric Wilcoxon pairwise test was then applied to compare alpha diversity between treatment groups and sampling times. The Benjamini-Hochberg method was applied to correct *P*-values for multiple comparisons [43]. Beta diversity was represented by a principal coordinate analysis of Bray-Curtis dissimilarities between samples [46]. Nonparametric analysis of similarities (ANOSIM) was also performed to study significant differences between microbial communities. The abundances of ARGs per antibiotic class between treatment groups and sampling times were also compared by implementing the nonparametric Wilcoxon pairwise test with the Benjamini-Hochberg correction method.

A multivariate association analysis was performed with the R Maaslin2 package [47] to identify differences in species abundances between treatment groups that showed significant resistome differences. From microeco abundance and taxonomy tables, this software allowed us to control for group as a fixed effect and to normalize the raw abundance dataset with the centered log-ratio (CLR) transformation method. Adjusted significance was computed with the Benjamini-Hochberg correction method (false discovery rate (FDR) < 0.05). The minimum prevalence and maximum significance were set at 0.15 and 0.05, respectively, and significant species abundance differences (*P*-value < 0.05) were calculated using the treatment group of interest as a reference.

## Results

### Weight and pathological outcome at the experimental farm

**Fig. 2:**
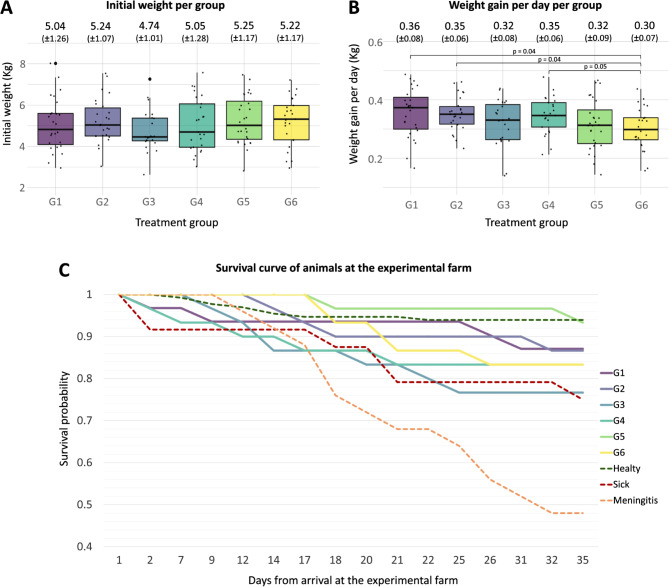
**(A)** Piglets’ weight distribution per treatment group at the start of the experiment. **(B)** Average piglet weight gain per day distribution per treatment group. **(C)** Survival curve analysis during the study period of treatment groups and clinical outcome of animals at the experimental farm. G1: trimethoprim/sulfamethoxazole, G2: colistin, G3: oral vaccination, G4: gentamicin, G5: untreated control with water acidification, G6: untreated control

The mean weight of the piglets before weaning was 5.10 kg (SD = 1.16 kg), showing a highly variable distribution with a coefficient of variation of 23%. Univariate analysis showed that weight variability was driven by the sow parity number, and significant differences were observed between litters. To avoid further biases, treatment groups were balanced according to weight by including one piglet per sow per treatment group, which excluded the sow effect. Pairwise comparisons of mean initial weights per group did not identify significant differences, as depicted in Fig. 2A.

The mean average daily weight gain (ADWG), excluding those animals that died before the end of the experiment, was 0.33 kg (SD = 0.076 kg) with a final mean weight of 19.05 kg (SD = 3.94 kg). The coefficients of variation of these parameters were also high (23 and 21%, respectively). Figure 2B shows pairwise comparisons of the ADWG between treatment groups and detected that piglets of the control group (G6, mean = 0.301 kg) had significantly decreased ADWG in comparison to the groups treated with antibiotics: trimethoprim/sulfamethoxazole (G1, mean = 0.355 kg, *P-adj* = 0.042), colistin (G2, mean = 0.352 kg, *P-adj* = 0.042), and gentamicin (G4, mean = 0.348 kg, *P-adj* = 0.050).

 A total of 47 piglets were transferred to the nursery pen at some point, either for weight loss and mild diarrhea or for clinical signs compatible with meningitis. Of them, 45 were treated with ceftiofur, and 17 died. Ten extra piglets suffered a sudden death. Animals’ identifiers per treatment and clinical information are summarized in Additional File: Table S4A. The vaccinated group (G3) exhibited the highest number of sick (*n* = 10) and dead (*n* = 7) animals compared to the other groups. The group with the lowest number of dead animals was the control group with water acidification (G5), that resulted in 2 deaths. Survival analysis identified that animals from the vaccinated group (G3) had 5.9-times higher risk of death (*P* = 0.042) in comparison to the animals from the control group with water acidification (G5) (Fig. 2C). A total of 25 animals suffered from meningitis, and 13 of them died. Statistical test showed a risk factor for death 5.6 times higher for animals that suffered meningitis compared to healthy animals (Fig. 2C). Macroscopic lesions from necropsies of dead piglets identified enteritis and pneumonia in 13 and 5 animals, respectively. The presence of both pathologies in the same individual was observed in three necropsies: two from the vaccinated group (G3) and one from the untreated control group (G6).

Antimicrobial resistance profiles for *E. coli* and *Streptococcus suis* were determined from seven necropsied animals belonging to the vaccinated group (G3, *n* = 4), the groups treated with trimethoprim/sulfamethoxazole (G1, *n* = 1), gentamicin (G4, *n* = 1), and the untreated control group (G6, *n* = 1). Animal identifiers and MIC values are detailed in Additional File: Table S4B. *E. coli* was isolated from all necropsied animals, being beta hemolytic the isolate obtained from the control group (G6). Coinfection with *S. suis* was found in three necropsied animals: one belonging to the group treated with trimethoprim/sulfamethoxazole (G1) and two to the vaccinated group (G3). All *E. coli* isolates were resistant to at least four antibiotics, with one of them being resistant to nine. Three isolates also exhibited Stb and EAST1 virulence factors. Regarding *S. suis*, two isolates were resistant to three antibiotics, while the other was resistant to five antibiotics. All three *S. suis* isolates were positive for the ST2/1–2 virulence factor.

### Sequencing quality analysis

After quality control, including host decontamination, trimming off adapters, and low-quality sequence removal from the shotgun metagenomic sequences of the 280 fecal samples, an average of 21.6 million reads were obtained, with a median of 24.3 million reads (Q1 = 16.4 M, Q3 = 27.0 M). Host contamination accounted for 13.02% (SD = 17.59%) of the trimmed reads after removal of adapters and low-quality sequences. Kraken2 software against the Maxikraken database at a 1% confidence level allowed taxonomic classification on an average of 61% (SD = 13.61%) reads per sample. Detailed information of sequencing data for all samples is available in Additional File: Table S2.

### Microbial diversity indexes

#### Diversity patterns between treatment groups

**Fig. 3:**
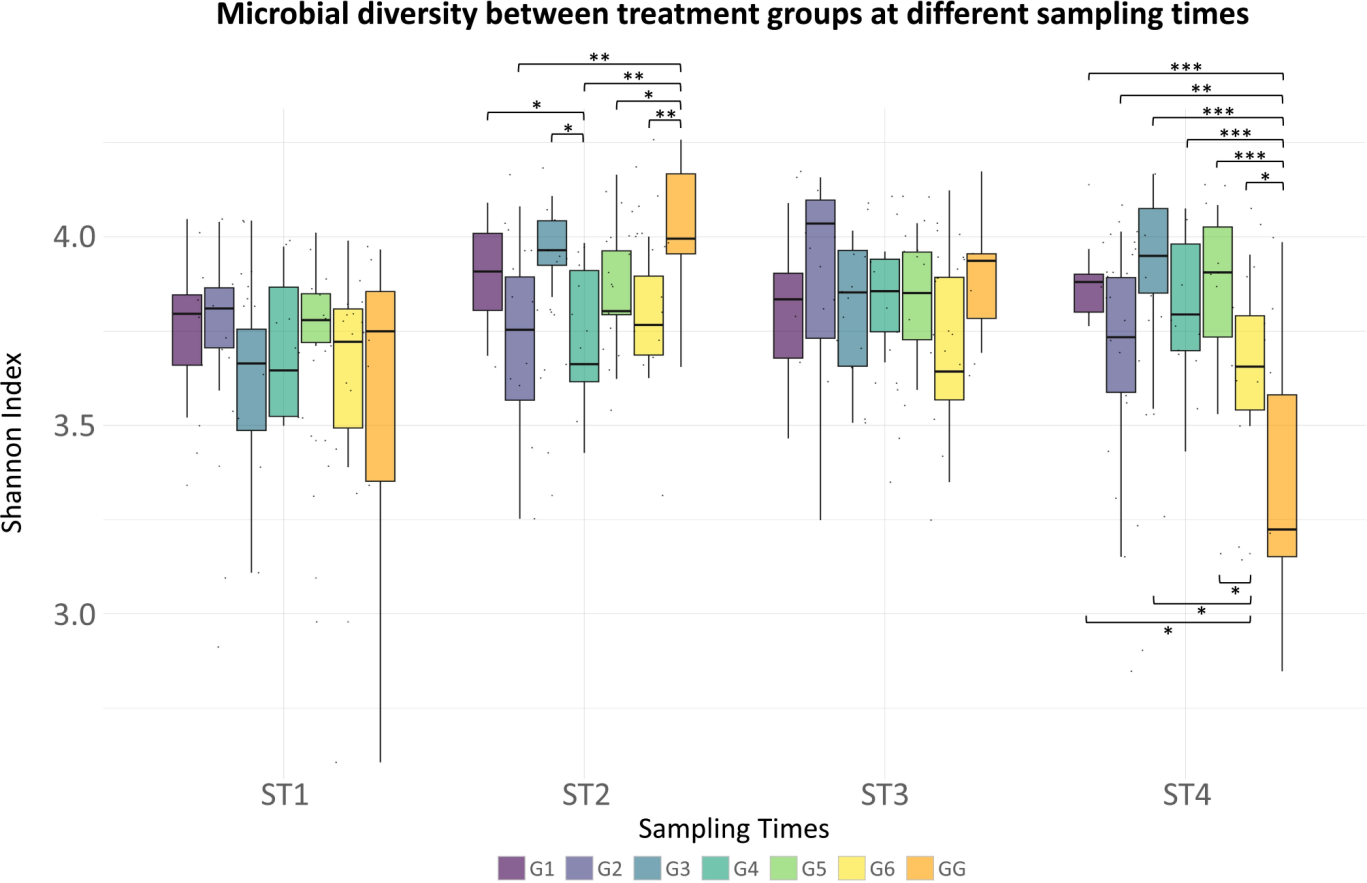
Shannon diversity represented by treatment group at the four different sampling times. Significant differences are highlighted at 0.01 (***), 0.05 (**), and 0.1 (*) significance levels. G1: trimethoprim/sulfamethoxazole, G2: colistin, G3: oral vaccination, G4: gentamicin, G5: untreated control with water acidification, G6: untreated control, GG: amoxicillin (farm of origin). ST1: one day before weaning, ST2: three days post-treatment, ST3: two weeks post-treatment, ST4: four weeks post-treatment

Shannon alpha-diversity indexes were compared between treatment groups taking into account all sampling times. Significantly lower microbial diversity was observed for the control group (G6) in comparison to the group treated with trimethoprim/sulfamethoxazole (G1, *P-adj* = 0.019), the vaccinated group (G3, *P-adj* = 0.025), and the control group with water acidification (G5, *P-adj* = 0.019). Shannon diversity indexes for alpha diversity were further analyzed to compare the treatments and control groups within the different sampling times. Figure 3 represents microbial diversity per treatment group at different sampling times, illustrating the main statistically significant differences at the 0.1, 0.05, and 0.01 significance levels. Kruskal-Wallis rank sum test identified heterogeneity in alpha diversity between groups at three days post-treatment (ST2, *P-adj* = 0.007) and four weeks post-treatment (ST4, *P-adj* = 0.002). Pairwise nonparametric t-tests comparing treatment and control groups two weeks post-treatment identified a significant increase in microbial diversity in the amoxicillin-treated group that remained at the farm of origin (GG) in comparison to the groups treated with colistin (G2, *P-adj* = 0.016), gentamicin (G4, *P-adj* = 0.016), the control group (G6, *P-adj* = 0.029), and the control group with water acidification (G5, *P-adj* = 0.079). Also at this timepoint, a significant lower microbial diversity was observed for the group treated with gentamicin (G4) in comparison to the groups treated with trimethoprim/sulfamethoxazole (G1, *P-adj* = 0.086) and colistin (G3, *P-adj* = 0.086). Conversely, four weeks after treatment, a significantly lower microbial diversity was observed in the amoxicillin-treated group that remained at the farm of origin (GG) compared to the group treated with trimethoprim/sulfamethoxazole (G1, *P-adj* = 0.006), colistin (G2, *P-adj* = 0.052), gentamicin (G4, *P-adj* = 0.006), the vaccinated group (G3, *P-adj* = 0.006), and the control group with water acidification (G5, *P-adj* = 0.006). Microbial diversity for the group that remained at the farm of origin treated with amoxicillin (GG) was also lower compared to the control group (G6, *P-adj* = 0.075). At this latter timepoint, a significantly lower microbial diversity was also observed for the control group (G6) in comparison to the group treated with trimethoprim/sulfamethoxazole (G1, *P-adj* = 0.056), the vaccinated group (G3, *P-adj* = 0.056), and the control group with water acidification (G5, *P-adj* = 0.087).

Analysis of variance of beta diversity, measured by Bray-Curtis dissimilarities after 999 permutations, did not detect significant differences between treatment groups (*P* = 0.434). Similar results were obtained with the ANOSIM test, (*R* statistic of 0.01 and *P* = 0.041) evidencing that microbial communities between treatment groups were not dissimilar.

#### Diversity patterns between sampling times

**Fig. 4:**
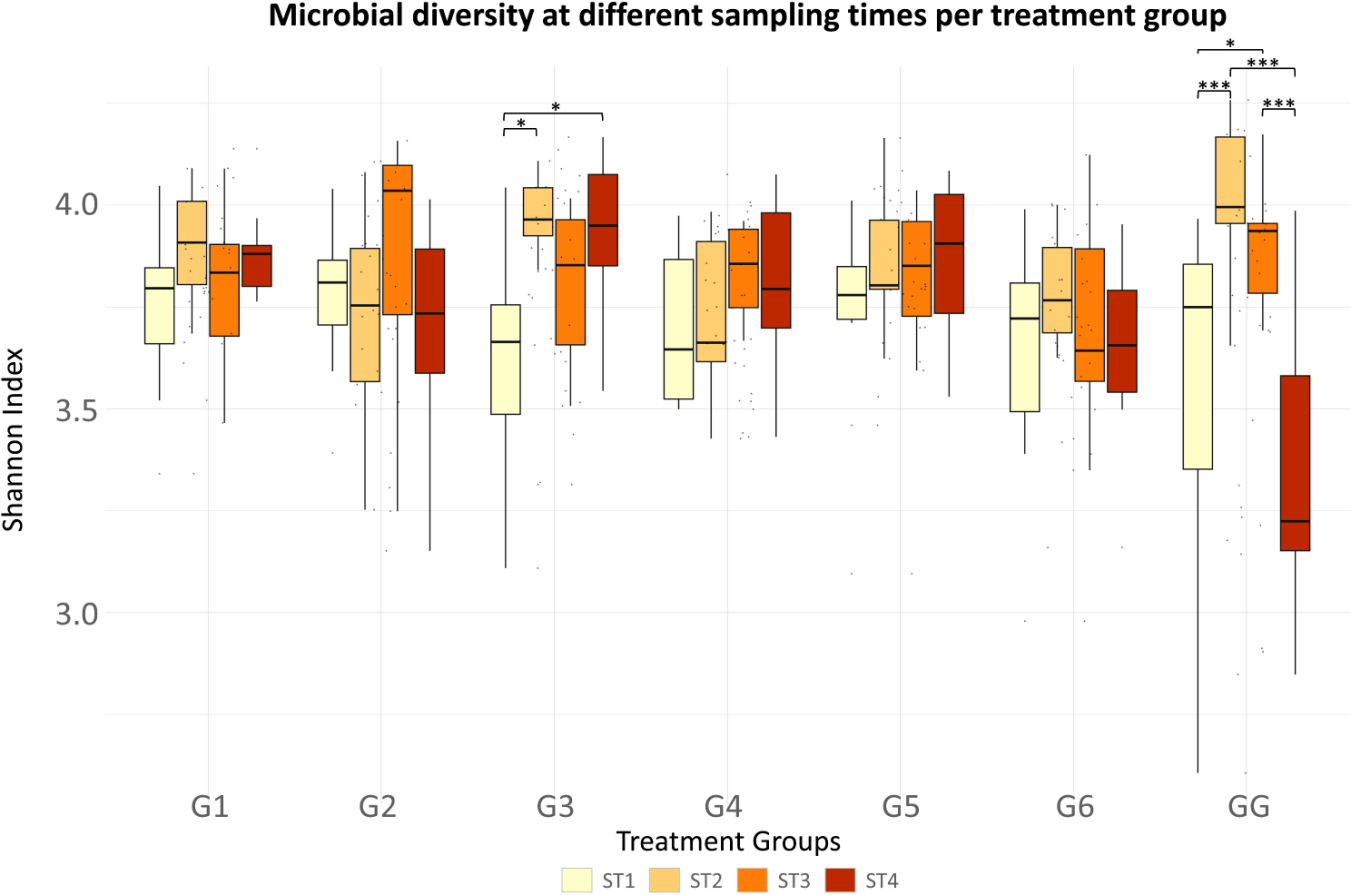
Shannon diversity represented by sampling time per treatment group. Significant differences are highlighted at 0.01 (***), 0.05 (**), and 0.1 (*) significance levels. G1: trimethoprim/sulfamethoxazole, G2: colistin, G3: oral vaccination, G4: gentamicin, G5: untreated control with water acidification, G6: untreated control, GG: amoxicillin (farm of origin). ST1: one day before weaning, ST2: three days post-treatment, ST3: two weeks post-treatment, ST4: four weeks post-treatment

Analysis of richness and evenness of all data between sampling times showed similar diversity patterns at one day before weaning (ST1) and four weeks post-treatment (ST4). In addition, a significantly lower diversity was observed one day before weaning when compared to the diversity observed after three days (ST2, *P-adj* = 0.001) and two weeks post-treatment (ST3, *P-adj* = 0.003). When comparing alpha diversity Shannon indexes across sampling times within groups (Fig. 4), the Kruskal-Wallis rank sum test identified differences in alpha diversity within the vaccinated group (G3, *P-adj* = 0.028) and within the amoxicillin-treated group that remained at the farm of origin (GG, *P-adj* = 0.000). Variations in alpha diversity indexes for the vaccinated group (G3) were observed at a 0.1 significance level when comparing microbial diversity before weaning (ST1) and three days (ST2, *P-adj* = 0.069) post-treatment. Regarding the group that remained at the farm of origin treated with amoxicillin (GG), these variations in alpha diversity showed an increase right after weaning that decreased to initial indexes by the end of the experiment. Significantly lower diversity was observed the day before weaning (ST1) and four weeks post-treatment (ST4) in comparison to the diversity estimated at three days post-treatment (ST2, *P-adj* = 0.003 and 0.002, respectively) and two weeks post-treatment (ST3, *P-adj* = 0.053 and 0.006, respectively).

Besides, Bray-Curtis dissimilarities to study the variance in beta diversity identified significant differences between sampling times (*P* = 0.001), meaning a time-specific effect with heterogeneous dispersion between them.

### Taxonomic composition and abundance patterns

**Fig. 5:**
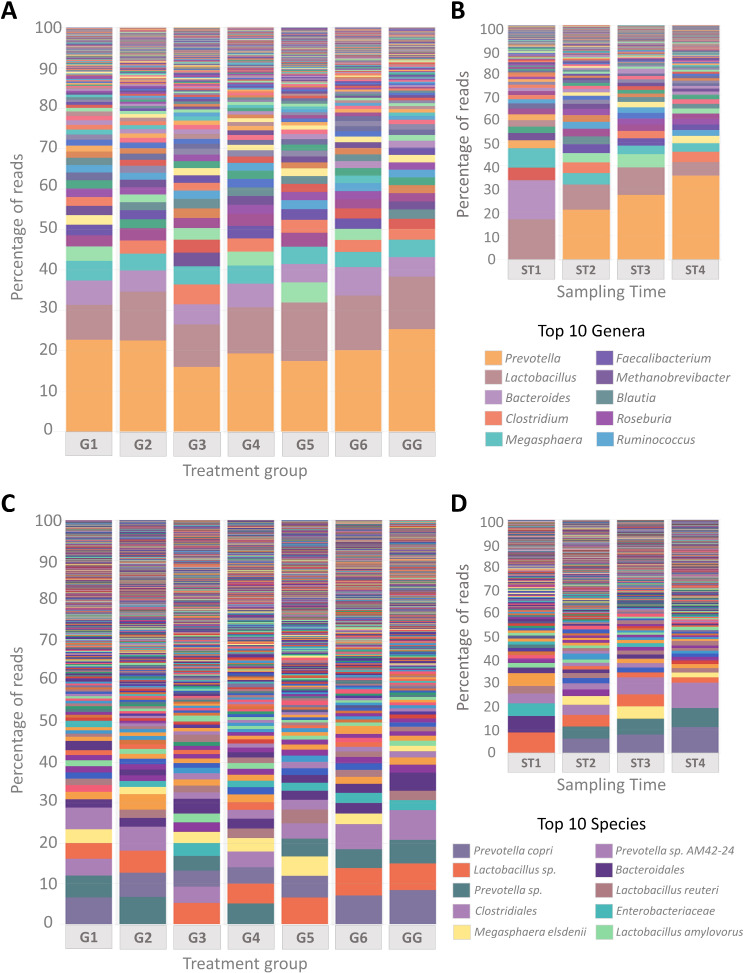
Percentages of relative abundances at the genus and species level out of the total taxonomically assigned reads by treatment group (**A**, **C**) and sampling time (**B**, **D**). G1: trimethoprim/sulfamethoxazole, G2: colistin, G3: oral vaccination, G4: gentamicin, G5: untreated control with water acidification, G6: untreated control, GG: amoxicillin (farm of origin). ST1: one day before weaning, ST2: three days post-treatment, ST3: two weeks post-treatment, ST4: four weeks post-treatment

The Phinch framework was used to represent the relative abundances of taxa by treatment group or by sampling time at the genus, species (Fig. 5) and phylum (Additional File: Figure S1) levels. Most abundant taxa at the phylum level were Bacillota and Bacteroidota, followed by Pseudomonadota, Actinomycetota, and Spirochaetota. Relative abundances were variable between treatment groups, but core phyla diversity did not show great differences (Additional File: Figure S1A). Regarding sampling times, increased relative abundances of Bacteroidota, Pseudomonadota, and Fusobacteriota were detected before weaning (ST1) compared to the relative abundances observed three days post-treatment (ST2), where Bacillota and Spirochaetota seemed to increase. Over time, the relative abundances of Bacillota were restored to the percentages observed the day before weaning, but Pseudomonadota and Spirochaetota did not change compared to the second sampling time (Additional File: Figure S1B).

At the genus level, the most abundant genera in all treatment groups were *Prevotella*, *Lactobacillus*, and *Bacteroides*, followed by *Clostridium* and *Megasphaera*. Again, core genera diversity between treatment groups did not show many differences, except for a decrease in the relative abundance of *Prevotella* in the vaccinated group (G3) and the control group with water acidification (G5) (Fig. 5A). However, further differences were identified when longitudinally comparing relative abundances at the genus level. Before weaning (ST1), an increase in the prevalence of *Lactobacillus*, *Bacteroides*, *Megasphaera*, and *Clostridium*, with the absence of *Prevotella*, was observed. However, after transition to the experimental farm and treatment, the prevalence of *Prevotella* increased over time, while the relative abundance of *Bacteroides*, *Lactobacillus*, and *Megasphaera* decreased (Fig. 5B). At the species level, most abundant taxa identified among the *Prevotella* genus was *P. copri* followed by *P.* AM42-24, although high number of *Prevotella* reads could not reach the species level or have not been yet identified. Similarly, the most abundant *Lactobacillus* species were *L. reuteri* and *L. amylovorus*, but other species of this genus may be found. The species *M. elsdenii* appeared to be the main taxa identified among the *Megasphaera* genus (Fig. 5C and 5D).

### Resistome characterization

Resfinder software identified a total of 229 different ARGs among samples, with a mean per sample of 45.54 ARGs (SD = 12.34 ARGs). AMR variants were represented by genes that confer resistance to 16 different drug classes, with aminoglycosides (*n* = 45), beta-lactams (*n* = 42), macrolides (*n* = 42), and tetracyclines (*n* = 37) being the most predominant. The total richness and abundance of ARGs per treatment group and sampling time are represented in Additional File: Figure S2. For raw and normalized PPMs values see Additional File: Tables S2 and S3, respectively. The richness of ARGs per treatment group did not show significant differences, but a higher richness of ARGs was observed at ST1 (before treatment) in all groups in comparison to the other sampling times (Additional File: Figure S2A).

A total of 2,150,769 reads covered the ARG sequence regions identified among samples. After normalization, the mean per sample was 346.29 PPMs (SD = 83.00 PPMs). Per antibiotic class, major normalized depth considering the sum of all samples was detected for genes conferring resistance to tetracyclines (*n* = 37,230.04 PPM), aminoglycosides (*n* = 25,571.23 PPM), and macrolides (*n* = 22,369.24 PPM), followed by beta-lactams (*n* = 5,132.44 PPM), and phenicols (*n* = 4,275.65 PPM) (Additional File: Figure S2B).

**Fig. 6:**
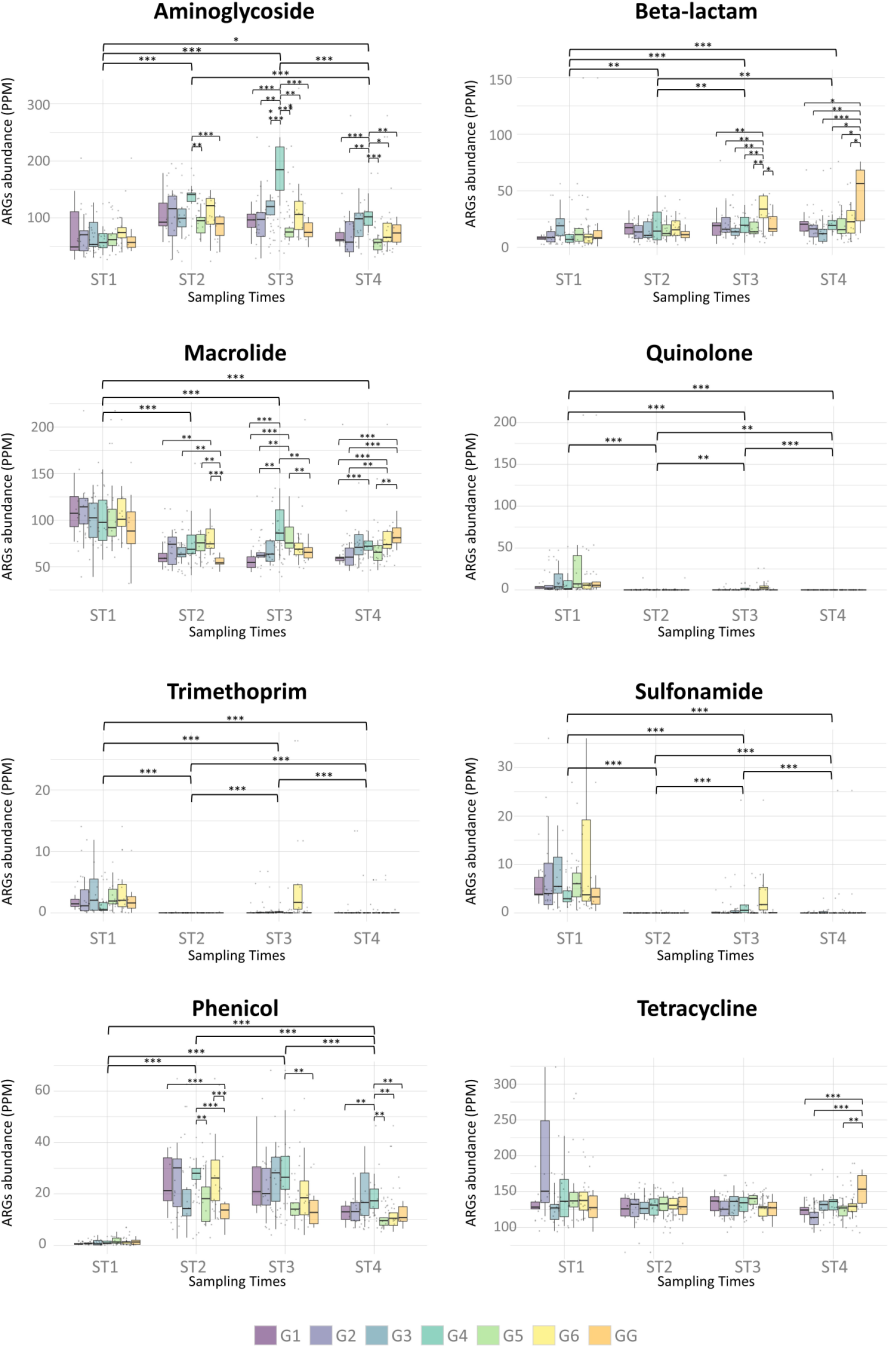
Abundance of ARGs to the different antibiotic classes represented in parts per million (PPM) of reads after normalization by treatment group and sampling times. Significant differences are highlighted at 0.01 (***), 0.05 (**), and 0.1 (*) significance levels. G1: trimethoprim/sulfamethoxazole, G2: colistin, G3: oral vaccination, G4: gentamicin, G5: untreated control with water acidification, G6: untreated control, GG: amoxicillin (farm of origin). ST1: one day before weaning, ST2: three days post-treatment, ST3: two weeks post-treatment, ST4: four weeks post-treatment

Figure 6 summarizes pairwise comparisons of normalized abundances of ARGs between treatment groups and sampling times. No significant differences were observed between treatment groups before weaning (ST1) for any antibiotic class. However, when comparing treatment groups three days (ST2), two weeks (ST3), and four weeks (ST4) post-treatment, significant differences were observed for tetracyclines, aminoglycosides, macrolides, beta-lactams, phenicols, sulfonamides, trimethoprim, and quinolones. For some antibiotic classes, ARG abundance in all groups diminished significantly after weaning and after treatment, as observed for macrolides, quinolones, trimethoprim, and sulfonamides (Fig. 6). The abundance of ARGs encoding macrolides was reduced from a mean of 105.00 PPMs (SD = 8.93 PPMs) before weaning-treatment (ST1) to a mean of 72.05 PPMs (SD = 11.14 PPMs) after treatment (ST2, ST3, ST4). However, significant differences were observed between treatment groups, with an increase in the abundance of ARGs to macrolides in the group treated with gentamicin (G4) two weeks after treatment (ST3, *P*-value < 0.05), and in the group that remained at the farm of origin treated with amoxicillin (GG) four weeks after treatment (ST4, *P*-value < 0.05), as depicted in Fig. 6. In contrast, the abundance of ARGs to phenicols significantly increased after treatment (ST2, ST3, ST4) in comparison to abundance levels before weaning (ST1, *P*-value < 0.01). The abundance of tetracycline resistance genes did not change significantly throughout the study, with an overall mean of 133.44 PPMs (SD = 12.87 PPMs). Significant differences were observed only four weeks after treatment (ST4), with a higher abundance of ARGs to tetracyclines in the group that remained at the farm of origin treated with amoxicillin (GG) in comparison to the groups treated with trimethoprim/sulfamethoxazole (G1, *P-adj* = 0.008), colistin (G2, *P-adj* = 0.004), and the control with water acidification (G5, *P-adj* = 0.012) (Fig. 6).

Focusing on the abundance of ARGs to aminoglycosides (Fig. 6), significant differences between treatment groups were observed at all sampling times after weaning (ST2, ST3, ST4). After three days post-treatment (ST2), a significant increase in the abundance of aminoglycoside resistance genes was observed in the gentamicin-treated group (G4) in comparison to the control group with water acidification (G5, *P-adj* = 0.012) and the group that remained at the farm of origin treated with amoxicillin (GG, *P-adj* = 0.000) (Fig. 6). After two weeks post-treatment (ST3), the increase in aminoglycoside resistance genes in the gentamicin-treated group (G4) was significant when compared to all treatment groups (*P*-value < 0.01). These differences remained significant four weeks post-treatment for all groups except for the vaccinated group (G3). Significant differences were also observed between treatment groups within sampling times when analyzing the abundance of ARGs to beta-lactams (Fig. 6). After two weeks post-treatment (ST3), the untreated control group (G6) showed a significant increase in the abundance of beta-lactam resistance genes compared to all treatment groups (*P*-value < 0.05, except for GG as *P-adj* = 0.052), which diminished after four weeks post-treatment (ST4). However, at this timepoint (ST4), an increase in ARG abundance for beta-lactams was observed for the group that remained at the farm of origin treated with amoxicillin (GG). Differences were significant when comparing abundances with the groups treated with colistin (G2, *P-adj* = 0.034) and the vaccinated group (G3, *P-adj* = 0.009). For the rest of the groups, differences were significant at a 0.1 significance level (G1 and G5 with *P-adj* = 0.059, and G4 and G6 with *P-adj* = 0.087) (Fig. 6).

### Differential abundance analysis at species level

**Fig. 7:**
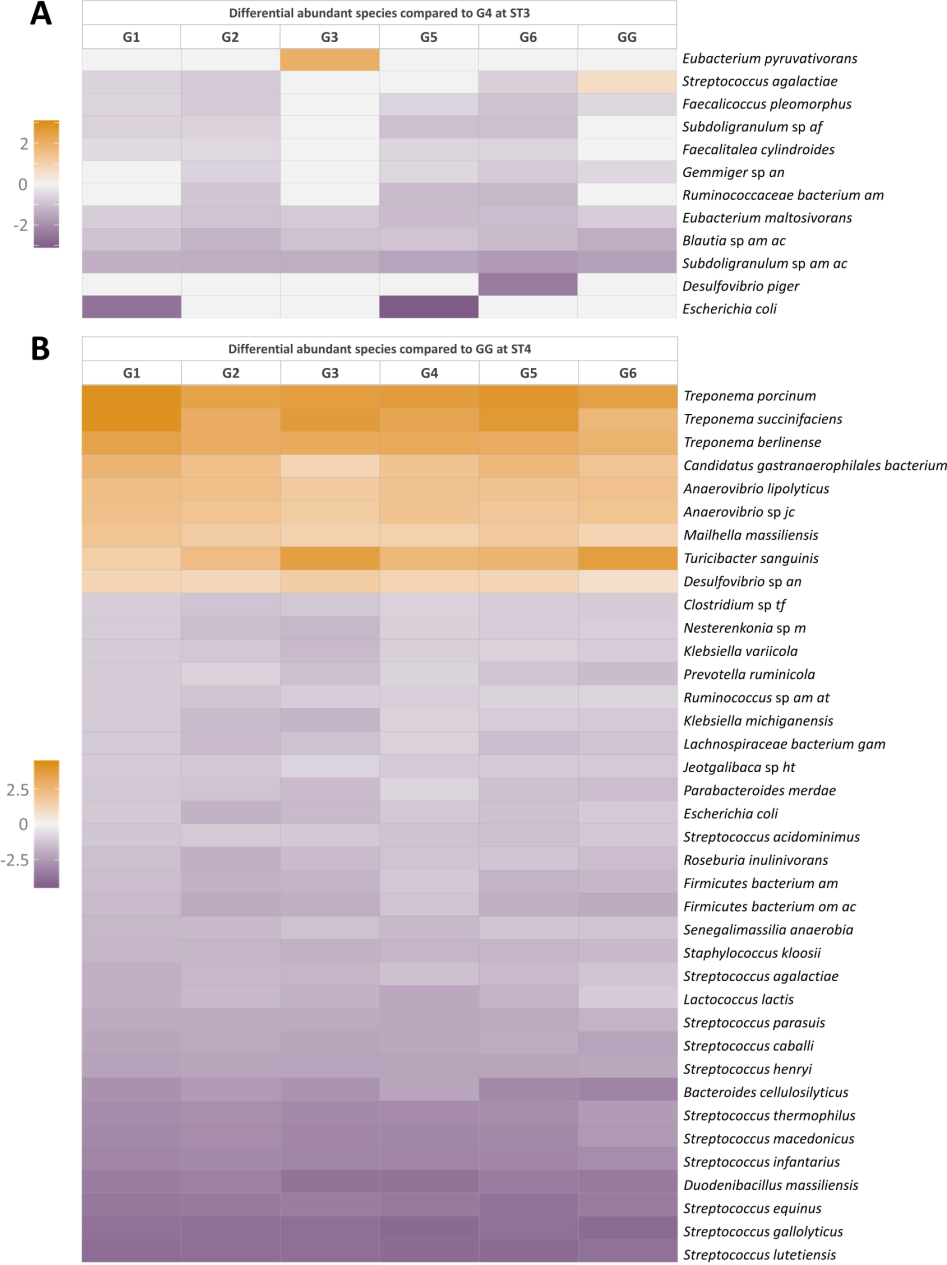
**(A)** Differentially abundant species with a significant *P*-value < 0.05 compared with the gentamicin-treated group (G4) two weeks after treatment (ST3). **(B)** Differentially abundant species with a significant *P*-value < 0.05 compared with the group that remained at the farm of origin treated with amoxicillin (GG) four weeks after treatment (ST4). G1: trimethoprim/sulfamethoxazole, G2: colistin, G3: oral vaccination, G4: gentamicin, G5: untreated control with water acidification, G6: untreated control, GG: amoxicillin (farm of origin)

A multivariate association analysis was performed at the species level for these groups and timepoints where significant differences (*P* < 0.05) were observed at the resistome level (i.e.,: the gentamicin-treated group (G4) at two weeks post-treatment (ST3) and the group that remained at the farm of origin treated with amoxicillin (GG) at four weeks post-treatment (ST4). Figure 7 summarizes the most relevant significant differences (± 0.5) in comparison to the other treatment groups. Due to the large number of differences for the group that remained at the farm of origin treated with amoxicillin (GG), only those differences common to all groups were included.

As depicted in Fig. 7A, the most significant differences were observed when comparing the gentamicin-treated group (G4, ST3) against the group that remained at the farm of origin treated with amoxicillin (GG, *n* = 104) and the control group (G6, *n* = 86), followed by the colistin-treated group (G2, *n* = 60). Beneficial species from the genera *Blautia* and *Subdoligranulum* and the species *Eubacterium maltosivorans* were underrepresented in all treatment groups at this sampling time in comparison to the gentamicin-treated group (G4). Moreover, *Desulfovibrio piger* was less abundant in the control group (G6). Similarly, *E. coli* was less represented in the group treated with trimethoprim/sulfamethoxazole (G1) and the control group with water acidification (G5). However, the species *Eubacterium pyruvativorans* and *Streptococcus agalactiae* were overrepresented in the vaccinated group (G3) and the group that remained at the farm of origin treated with amoxicillin (GG), respectively.

When further analyzing the group that remained at the farm of origin treated with amoxicillin (GG) at four weeks post-treatment (ST4), significant differences at the species level were observed (Fig. 7B), especially when compared with the vaccinated (G3, *n* = 776), the control with water acidification (G5, *n* = 526), and the untreated control (G6, *n* = 504) groups. For all treatment groups, increased abundance in species such as *Treponema* (*T. porcinum*, *T. succinifaciens*, and *T. berlinense*) and *Anaerovibrio* were detected compared with the farm of origin treated with amoxicillin (GG). Other underrepresented species in the group that remained at the farm of origin treated with amoxicillin (GG) were the beneficial *Mailhella massiliensis*, *Turicibacter sanguinis*, and several species of *Desulfovibrio*. Also, the novel species of cyanobacteria *Gastranaerophilales bacterium* of the Melainabacteria phylum was underrepresented in this group (GG). In addition, an increase in the abundance of many potential opportunistic pathogens of the *Streptococcus* genera was observed in this group (GG), likewise, an overrepresentation of the species *Duodenibacillus massiliensis* and *Lactococcus lactis* was also detected (Fig. 7B).

## Discussion

Post-weaning diarrhea is one of the major threats to piglets’ health during the weaning stage. This transition period is characterized by many physiological and environmental stressors that are crucial for pig performance and consequently for the swine industry [48, 49]. The commercial vaccine and antibiotic treatments applied in this study are commonly used to prevent and treat post-weaning-related infections, diminishing disease incidence and mortality. In addition, the implemented shotgun metagenomics approach allowed an in-depth analysis of the pig gut microbiome and resistome diversity and abundance, which enabled intra- and intergroup comparative analyses. It should be mentioned that the fact that no significant differences were observed between treatment groups before weaning in any of the analyses performed in this study warranted that groups were properly balanced at the start of the experiment. Therefore, ensuring that the posteriori observed differential patterns were mediated mainly by the use of antibiotics.

The effect of antibiotics as growth-promoters has been widely described for more than eighty years, and since then, they have been applied in pig production to prevent disease and increase productivity. Although these practices are already banned in European countries, there is a growing concern regarding their consequence on the emergence of antimicrobial resistance [50]. Herein, this growth-promoting effect is demonstrated again, showing an increase in average daily weight in the different antibiotic-treated groups compared to the untreated control group. However, it is interesting to note that differences were not significant when comparing the antibiotic-treated groups to the vaccinated group or the control with water acidification. These results suggest that other alternatives may have the same positive effect when considering the maintenance of piglet weight and productivity. Besides, survival analyses showed that the control group with water acidification was the group that performed better at the experimental farm, being significantly different from the vaccinated group, which surprisingly showed the highest number of sick and dead animals. Therefore, our results suggest that the oral commercial vaccine in this experimental setting did not have a protective effect against *E. coli*. Additionally, it may indicate that PWD symptomatology observed in this study could not be correlated to ETEC proliferation but to other predisposing factors. Regarding the group that remained at the farm of origin, amoxicillin treatment seemed to prevent post-weaning diarrhea or clinical signs compatible with *S. suis*.

On the other hand, the longitudinal microbial diversity analyses showed a similar pattern in all treatment groups, characterized by a significant increase in microbial diversity right after weaning that diminished to initial diversity levels four weeks after treatment. Therefore, the increase in microbial diversity after weaning appears not to be explained by a prompt effect of the antibiotic treatments. Presumably, this increase in diversity may be influenced by the removal of sow milk and lactogenic maternal immunity, and the transition to a solid diet with a larger proportion of fiber. These changes in weaning can affect food intake, piglets’ acquired immunity, and susceptibility to pathogens [51–53]. However, significant differences between sampling times were observed only for the group that remained at the farm of origin treated with amoxicillin, which correlated with an increased diversity variability in comparison to the rest of the groups. Interestingly, at the experimental farm, no significant differences between treatment groups were observed until four weeks post-treatment. At this time, the significant decrease in microbial diversity in the group that remained at the farm of origin treated with amoxicillin, and, to a lesser extent, the untreated control group could suggest an impairment in the piglet’s gut microbiome at the end of the experiment and in the long-term, which may have a direct influence on the host immune system [54, 55]. In addition, regarding the group that remained at the farm of origin treated with amoxicillin, this disruption of the microbial communities is most likely associated with the continuous amoxicillin treatment implemented in the farm that may be selecting for specific microbial communities due to prolonged antibiotic pressure [55]. The fact that this decrease in microbial diversity is not observed in the antimicrobial-treated groups at the experimental farm could be explained by the shorter prescription period of the treatments.

The microbiome diversity variations observed immediately after weaning may be the explanation for the differences identified in the abundance of resistance genes to specific antibiotic classes after weaning [56]. Despite alpha diversity indexes not being significantly different before and after treatment in most treatment groups, clear microbial shifts were observed at phylum, genera, and species levels that can be explained by changes in the diet and the environment during the transition [57]. These changes in the microbial composition may influence the decrease of specific microorganisms carrying AMR genes for macrolides, quinolones, trimethoprim, and sulfonamides, which could explain the higher abundance of these specific AMR genes before weaning. However, the increase in resistance genes to phenicols immediately after weaning in all treatment groups may indicate an increase in certain species harboring these genes. The fact that significant differences in AMR gene abundance were observed only in the gentamicin-treated group and the group that remained at the farm of origin treated with amoxicillin, suggests that different treatments for PWD have a different influence on the emergence and selection of AMR genes [58, 59]. Treatments with trimethoprim/sulfamethoxazole and colistin appeared to have no effect on the abundance of AMR for any of the antibiotic classes. Also, no specific genes conferring resistance to these antibiotic classes were identified after treatment, meaning that no selective pressure occurred for these antibiotics in this experimental setting. Similarly, oral vaccination and acidifiers in water appeared to have no influence on the selection of AMR. Besides, the gentamicin treatment applied in this study showed an effect not only in the increase in abundance of AMR genes for aminoglycosides but also for macrolides. In the same way, the amoxicillin treatment applied at the farm of origin seemed to have an impact not only on the emergence of AMR genes to beta-lactams, but also to tetracyclines. Changes in the abundance of certain species during treatment, as demonstrated by the multivariate analyses, may indicate acquisition of specific AMR genes by these species. Differences observed for the group that remained at the farm of origin treated with amoxicillin four weeks after the start of the treatment showed an overrepresentation of *Streptococcus* species that have been previously associated with mastitis (*S. lutetiensis* and *S. agalactiae*), endocartidis (*S. gallolyticus*), and respiratory infections (*S. parasuis*), and *Duodenibacillus massiliensis*, which has been associated with patients suffering from iron-deficiency anemia [60–64]. However, an increase in the abundance of *Lactococcus lactis* was also detected, which has been studied as a probiotic to reduce pathogen infection during the post-weaning period [65]. Further studies with better taxonomy resolution combining long and short-read sequencing technologies may allow a better understanding of microbial communities at the species level and their associated AMR genes.

Overall, comparative analyses on the diversity and abundance of the microbiome and resistome during post-weaning indicate that continuous treatment with amoxicillin as implemented at the farm of origin have a negative effect on the pig gut microbiome, reducing microbial diversity and increasing the emergence of AMR genes. Therefore, the increase in abundance for the species *Treponema porcinum*, *Treponema succinifaciens*, and *Turicibacter sanguinis* in all groups in comparison to the group that remained at the farm of origin treated with amoxicillin should be further investigated. Moreover, treatment with gentamicin seemed to contribute to both impaired microbiota and the emergence of AMR genes, shortly after treatment. Furthermore, considering the poorer clinical outcome of the vaccinated group in our study, our results suggest that non-antibiotic alternatives, such as acidifiers in the water, offer a good alternative helping to develop a balanced gut microbiome and reduced susceptibility to pathogens during weaning and post-weaning stages, also having a positive effect on growth performance, viability, and productivity while reducing the emergence of antimicrobial resistance.

## Conclusions

Overall, the growth promoting effect of antimicrobials was again demonstrated, although differences were not significant when comparing to the non-antibiotic alternatives of vaccination and water acidification. Additionally, a prolonged antibiotic treatment not only has a deleterious effect on microbial diversity and composition, but also in the emergence of antimicrobial resistance. Altogether, our results suggest that non-antibiotic alternatives, such as supplementing drinking water with acidifiers, are able to improve survival without compromising growth performance, microbial diversity and composition, and its resistome.

### Electronic supplementary material

Below is the link to the electronic supplementary material.

Supplementary Material 1

Supplementary Material 2

Supplementary Material 3

## Data Availability

Metagenomic raw data have been submitted to the NCBI's sequence read archive with the BioProject accession number PRJNA1010706.
