# Supplementary figures and images for: Gut microbiome and resistome characterization of pigs treated with commonly used post-weaning diarrhea treatments
Source: Anim Microbiome. 2024 May 3;6:24. doi: 10.1186/s42523-024-00307-6 (PMC11067243; doi:10.1186/s42523-024-00307-6)

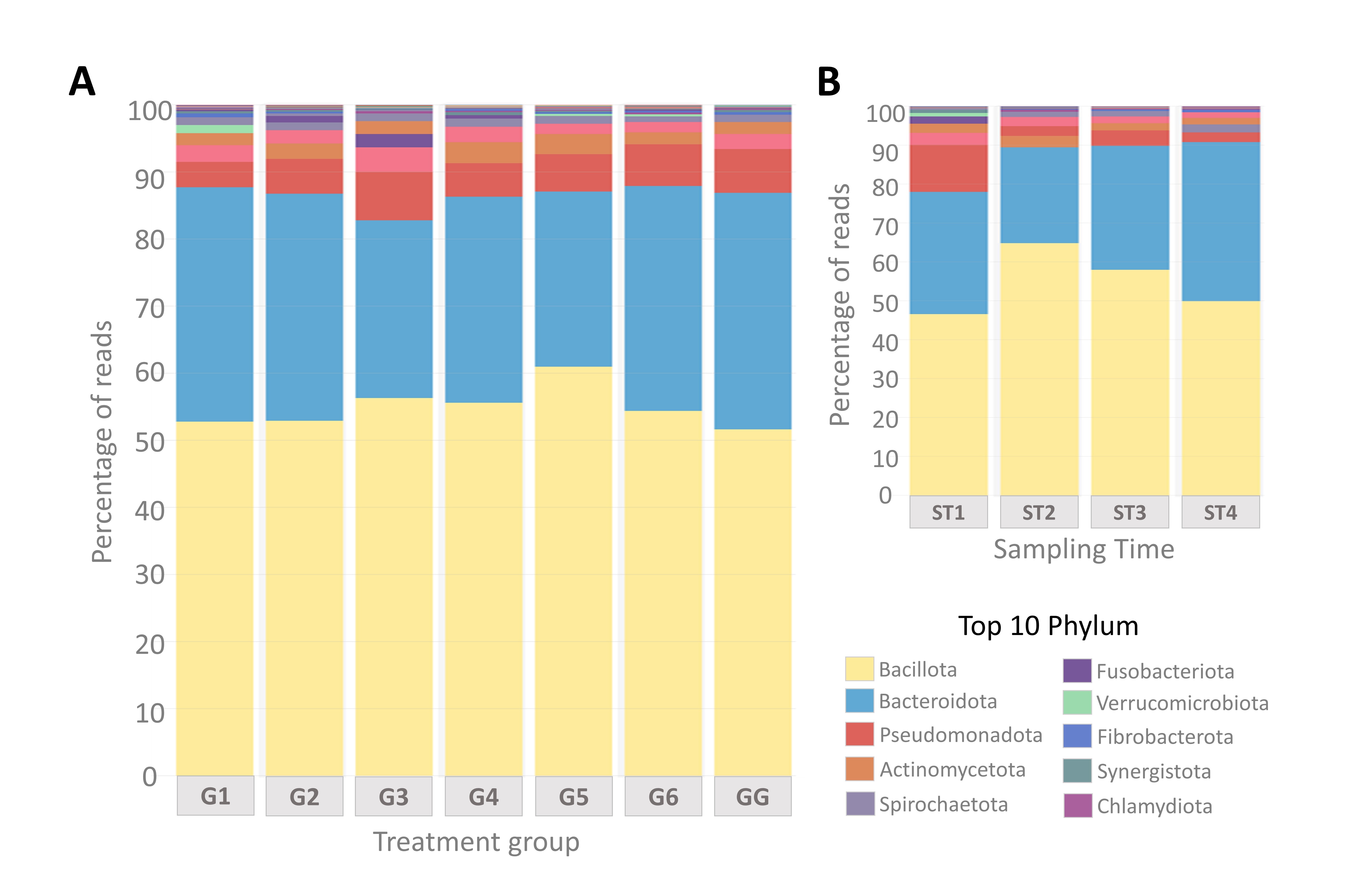

Supplement: Supplementary file 2 — Supplementary Material 2 [file 42523_2024_307_MOESM2_ESM.jpg]

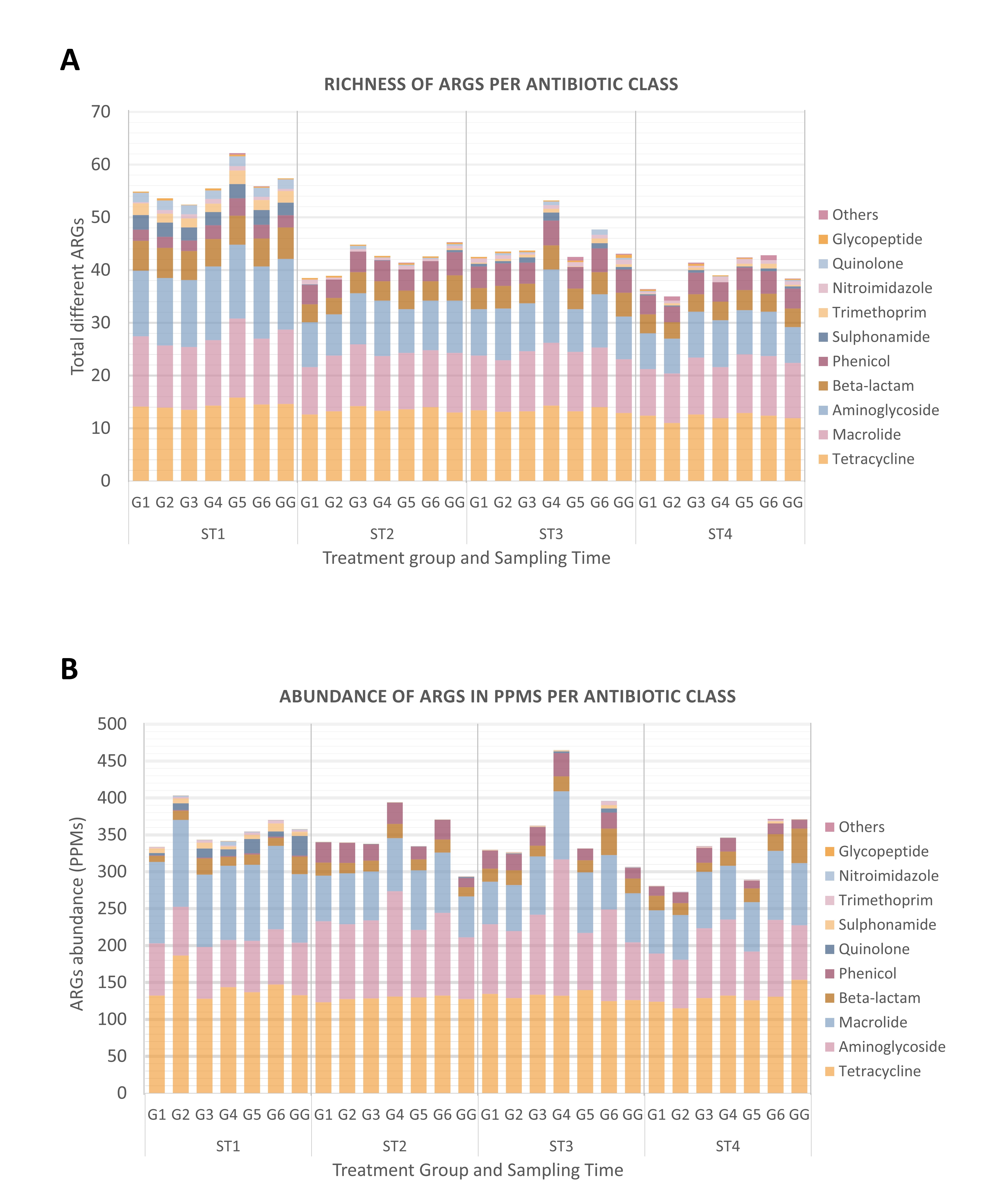

Supplement: Supplementary file 3 — Supplementary Material 3 [file 42523_2024_307_MOESM3_ESM.jpg]
